# Targeting the Nuclear Export Protein XPO1/CRM1 Reverses Epithelial to Mesenchymal Transition

**DOI:** 10.1038/srep16077

**Published:** 2015-11-05

**Authors:** Asfar S. Azmi, Irfana Muqbil, Jack Wu, Amro Aboukameel, William Senapedis, Erkan Baloglu, Aliccia Bollig-Fischer, Gregory Dyson, Michael Kauffman, Yosef Landesman, Sharon Shacham, Philip A. Philip, Ramzi M. Mohammad

**Affiliations:** 1 Department of Oncology, Wayne State University School of Medicine, Detroit MI 48201; 2 Karyopharm Therapeutics Newton Massachusetts, USA; 3 iTRI Hamad Medical Corporation, Doha Qatar.

## Abstract

Here we demonstrate for the first time that targeted inhibition of nuclear exporter protein exportin 1 (XPO1) also known as chromosome maintenance region 1 (CRM1) by Selective Inhibitor of Nuclear Export (SINE) compounds results in reversal of EMT in snail-transduced primary human mammary epithelial cells (HMECs). SINE compounds selinexor (KPT-330) and KPT-185, leptomycin B (LMB as +ve control) but not KPT-301 (–ve control) reverse EMT, suppress mesenchymal markers and consequently induce growth inhibition, apoptosis and prevent spheroid formation. SINE treatment resulted in nuclear retention of snail regulator FBXL5 that was concurrent with suppression of snail and down-regulation of mesenchymal markers. FBXL5 siRNA or transfection with cys528 mut-Xpo1 (lacking SINE binding site) markedly abrogated SINE activity highlighting an XPO1 and FBXL5 mediated mechanism of action. Silencing XPO1 or snail caused re-expression of FBXL5 as well as EMT reversal. Pathway analysis on SINE treated HMECs further verified the involvement of additional F-Box family proteins and confirmed the suppression of snail network. Oral administration of selinexor (15 mg/kg p.o. QoDx3/week for 3weeks) resulted in complete cures (no tumor rebound at 120 days) of HMLER-Snail xenografts. These findings raise the unique possibility of blocking EMT at the nuclear pore.

The majority of cancer patients with advanced or metastatic disease have limited long-term benefits from conventional cytotoxic and targeted drugs. In most instances, metastasis develops by the aberrant revival of an embryonic developmental program termed as epithelial-to-mesenchymal transition (EMT)^1^. EMT is an intricate process where cancer cells demonstrate the loss of polarity and change their morphology from epithelial to mesenchymal. Such morphological changes allow the cells to attain plasticity thereby enhancing their motility, invasiveness, and ultimately rendering them metastatic^2^. EMT is orchestrated by numerous proteins that are uniquely placed in different sub-cellular compartments of the cell^3^. Investigations in the last few years have helped in the better understanding of the many diverse EMT stimulating transcription factors (TFs), along with enhanced understanding of their compartmentalization dependent regulation in cancer cells^4^.

A majority of EMT promoting proteins and TFs including snail are well known cargoes of the nuclear-cytoplasmic transporters: karyopherins^5^. The karyopherins, are divided into two major classes i.e. importins and exportins. The importin alpha is a nuclear importer of nuclear localization signal sequence (NLS) harboring cytosolic proteins^6^. On the other hand, the export of major EMT promoting TFs is undertaken solely by Exportin1/XPO1 [chromosome maintenance region 1 (CRM1)] that recognizes a hydrophobic, nuclear export sequence (NES)^7^. More significantly, earlier studies have clearly demonstrated that aside from regulation at the transcriptional level, the activity of different TFs has been proposed to be modulated through mislocalization within the cell thereby causing profound impact on the cellular signaling^8^. Given that disturbed protein transport mechanisms are quite commonly observed in cancer^9^, this phenomenon certainly points to the critical role of nucleocytoplasmic transport in the biology of EMT.

Snail, is a TF that is a negative regulator of epithelial morphology promoter E-cadherin and has been extensively studied for its role in EMT^10^. As such, snail is a very unstable protein and is recognized to undergo a rapid turnover^11^. Snail is regulated by a number of different post-translational mechanisms such as ubiquitination, phosphorylation, and lysine oxidation^12^. These post-translational control mechanisms have been shown to affect snail stability, function as well as its sub-cellular localization^13^. Two major RING finger ubiquitin ligases that belong to the Skp1-Cullin-Rbx1-F-box (SCF) F-Box family are recognized to influence snail’s proteasomal breakage dependent regulation mechanisms. SCF^β-TrCP1/FBXW1^ has been shown to polyubiquitinate snail once it is phosphorylated by GSK-3β^14^. The F-Box family members FBXL5^15^ and FBXO11^16^ have been recognized as nuclear snail regulators. These multiple lines of evidence quite clearly support the notion that protein localization dependent destabilization of snail regulators can certainly impact snail stability leading to modulation of EMT.

It is well recognized that nuclear export proteins, particularly XPO1, are deregulated in cancer^17^. Nevertheless, until now there are no published studies reporting on how abnormal nuclear export may influence EMT signaling. In this direction, we have demonstrated that inhibition of XPO1 by Selective Inhibitor of Nuclear Export (SINE) compounds induce the nuclear localization of F-Box protein FBW7^18^. This leads to nuclear degradation of well recognized EMT promoter notch, concordant with apoptosis induction in pancreatic cancer cells. Building on these findings, here we evaluate the potential for EMT-reversing ability of SINE compounds in snail-transduced primary human mammary epithelial cells in the context of F-Box proteins transport mechanisms.

## Results

### SINE compounds reverse EMT leading to growth inhibition and apoptosis induction in Snail-transduced HMECs

**Figure 1:**
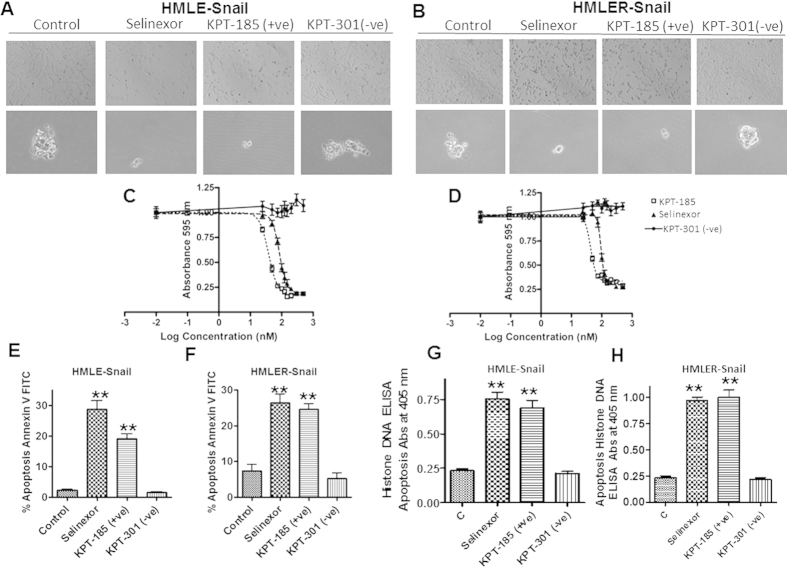
XPO1 inhibition by Specific Inhibitor of Nuclear Export (SINE) compounds blocks EMT. (**A**,**B** top panels) XPO1 inhibition reverses EMT. HMLE-snail and HMLER-snail cells were grown (50,000 cells/well in six well-plates) for 24 hrs followed by exposure to different SINE compounds and inactive analog KPT-301 (at 1 μM each) for another 24 hrs. Photomicrographs (40× magnification) showing elongated cells in the control and circular cells in the treated groups. KPT-301 (−ve control) does not induce any change in morphology of either HMLE-Snail or HMLER-Snail. Pictures representative of three independent experiments (**A**,**B** lower panel) SINE not inactive analog [150 nM] once a week for two weeks disrupt HMLE spheroid formation. Spheroids are representative of three independent experiments (**C**,**D**) Growth inhibition (MTT assay 72 hrs) by SINE compounds selinexor, KPT-185 (+ve control) not by inactive analog KPT-301. Graphs representative of three independent experiments with six replicates per dose presented. (**E**,**F**) SINE not inactive analog induce apoptosis in HMLE-snail and HMLER-snail cells. 50,000 cells were grown in six well plates in triplicate overnight followed by exposure to (150 nM) of SINE and –ve control KPT-301 for 72 hrs followed by Annexin V FITC analysis (according to manufacturer’s protocol BD Biosciences, USA). Apoptosis images representative of three independent experiments. (**G,H**) Apoptosis analysis in HMLE-snail and HMLER-snail by Histone DNA ELISA (Roche) under similar treatment conditions. **p < 0.01 when compared to control.

In this work, we have selected the HMECs since EMT is induced with the transduction of a single gene (i.e., snail giving HMLE-snail cells) thereby allowing a very compact cellular model to specifically study the role of nuclear transport on snail induced EMT pathways. As shown in Fig. 1A,B (top panel), SINE compounds (Selinexor and KPT-185 but not KPT-301 as –ve control) treatment (1 μM for 24 hrs) resulted in the reversal of the mesenchymal phenotype to epithelial phenotype (MET) [circular (epithelial) cells in treated group vs elongated (mesenchymal) cells in control or inactive (KPT-301) analog HMLE-snail as well as HMLER-snail cell lines (ras and snail tranduced cells: HMLER-snail). Similar results were obtained with a natural product type XPO1 inhibitor leptomycin B (LMB) used as positive control (Supplementary Fig. 1) or through siRNA silencing of CRM1 and snail (Supplementary Fig. 2). Of importance is the observation that MET occurs at 3 hrs (a time point where no cell apoptosis is observed Supplementary Fig. 3). Supplementary videos S1 Control, S2 selinexor treated, S3 KPT-185 treated and S4 LMB treatment at 3 hrs showing changes in morphology from mesenchymal to epithelial.

Given that SINE compounds are developed as anti-cancer agents, we evaluated their activities against HMECs using multiple cytotoxicity assays at long term exposure. As shown in Fig. 1A,B (lower panels), active SINE compounds, but not the inactive analog, caused disruption of spheroids (p < 0.001 see also Supplementary Fig. 4). The observed reversal of EMT and spheroid disruption by SINE compounds was concurrent with growth inhibition as determined by MTT assay (Fig. 1C,D IC_50s_ < 100 nM for HMLE-snail and HMLER-snail) and suppression of colony formation (Supplementary Fig. 5). At late time points (72 hrs) we observed apoptosis induction as verified by Annexin V FITC (Fig. 1E,F) in HMLE-snail and HMLER-snail respectively. That SINE compounds have some cancer selectivity was proven by the lack of statistically significant apoptosis in normal MCF-10ANeoT by analog KPT-185 (150 nM) at 72 hrs (Supplementary Fig. 3B). Histone DNA ELISA assay were in line with the Annexin apoptosis results and we observed statistically significant apoptosis induction by selinexor and KPT-185 (**p < 0.001) and not the inactive analog (Fig. 1G,H for HMLE-snail and HMLER-snail respectively).

### SINE compounds suppress EMT markers

**Figure 2:**
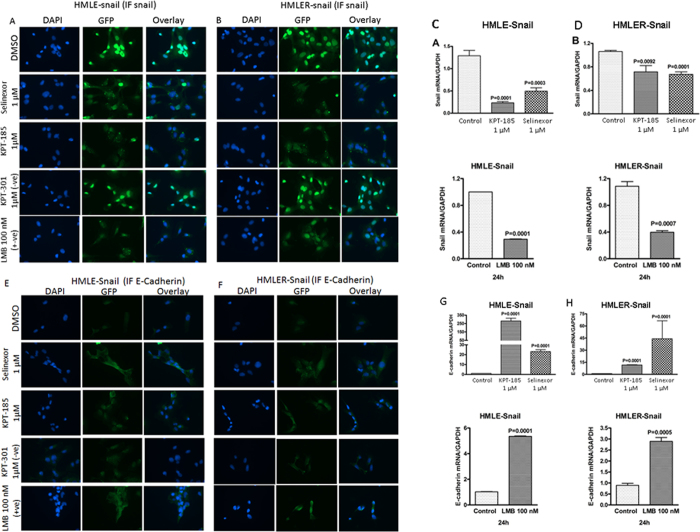
SINE compounds suppresses EMT markers and induces nuclear degradation of snail in HMLE-snail models. Cells were grown at a density of 3000 cells per well in 4 well chambered slides. After 24 hrs the cells were exposed to indicated concentrations of SINE, LMB or KPT-301 (−ve control) and IF was performed as described in methods section. (**A**,**B**) Snail IF images of HMLE-snail and HMLER-snail cells exposed to 1 μM concentrations of either selinexor, KPT-185 or KPT-301(–ve control) for 24 hrs (40 ×). Note: Images showing reduction in snail upon selinexor, KPT-185, or LMB (100 nM for 24 hrs as +ve control) exposed cells that was absent in KPT-301 treatment group (used as −ve control). Data representative of three independent experiments. (**C**,**D**) HMLE-snail and HMLER-snail cells were grown in six well plates in duplicate at a density of 50,000 cells per well overnight and then exposed to either selinexor or KPT-185 for 24 hrs. RNA was isolated and RT-PCR was performed as described in methods section. Note suppression of snail mRNA in both cell models tested. Similar results were obtained by +ve control LMB exposure (100 nM for 24 hrs) (**C,D** lower panels). Data representative of two independent experiments (**E**,**F**) Immunofluorescence images (40×) showing enhancement in E-cadherin under similar treatment conditions. (**G**,**H**) RT-PCR analysis for E-Cadherin expression under similar treatment conditions. Data is representative of two independent experiments.

**Figure 3:**
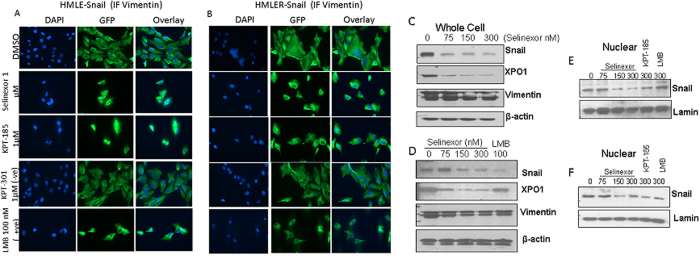
Molecular analysis of SINE compounds in HMECs. HMLE-snail and HMLER-snail cells were grown (3000 cells per well) in chambered slides. Cells were exposed to indicated doses of SINE, LMB or KPT-301 as –ve control and immunofluorescence assay was performed a described in methods. (**A,B**) Immunofluorescence images [40×] showing suppression of cytosolic vimentin upon treatment with the drugs in HMLE-snail and HMLER-snail respectively. (**C,D**) Western blotting of whole cell lysates from HMLE-Snail (top) and HMLER-Snail (lower) cells exposed to indicated concentration of SINE compounds or LMB for 72 hrs. Blots were probed for CRM1 (Santa Cruz, USA), vimentin (Santa Cruz, USA), Snail (Cell Signaling, Danvers USA) and β-actin (Sigma, USA) as a loading control. (**E,F**) Nuclear lysates isolated from HMLE-snail (top) or HMLER-snail (bottom) post SINE treatment under similar conditions (for 24 hrs). Blots were re-probed with lamin for nuclear loading control. Note: in both cellular models, there is suppression of nuclear snail protein by selinexor and +ve control KPT-185 or LMB. Blots representative of three independent experiments.

Snail is recognized to be an export target of XPO1 protein^19^. In order to evaluate the nuclear localization changes of different EMT-related markers, immunofluorescence analyses were performed. Results of Fig. 2A,B, demonstrate that SINE treatment for 24 hrs caused reduction in snail in both cellular models. LMB showed similar reduction in snail (Fig. 2A,B lowermost panels). Additional conclusive evidence came from RT-PCR analysis where we observed statistically significant suppression of snail by selinexor, KPT-185 or LMB (Fig. 2C,D). Reduction in snail was concurrent with the enhancement of E-cadherin as detected by immunofluorescence assay (Fig. 2E,F) and through RT-PCR analysis where statistically significant enhancement of E-cadherin was observed upon drug treatment (Fig. 2G,H). We also observed marked reduction of total and cytosolic vimentin upon SINE or LMB treatment in HMLE-snail and HMLER-snail cells (Fig. 3A,B). The inactive analog KPT-301 did not induce any substantial changes in vimentin or snail (Fig. 3A,B lower panels). In order to further confirm our results, western blotting was performed. Result of Fig. 3C (for HMLE-snail) and D (for HMLER-snail) show that SINE compound treatment caused suppression of snail, vimentin and XPO1/CRM1^20^. LMB showed similar inhibition of these markers (Fig. 3D far right lane). In order to confirm the immunofluorescence results, we also evaluated the nuclear expression levels of snail in the nuclear fraction of the HMLE-snail and HMLER-snail cells. As can be seen in Fig. 3E (for HMLE-snail) and F (HMLER-snail), SINE (Selinexor, KPT-185) or LMB treatment resulted in nuclear reduction in snail protein as well. Supporting evidence came from siRNA studies where we observed statistically significant enhancement in E-cadherin mRNA expression in CRM1 silenced cells (Supplementary Fig. 6A). Conversely, siRNA silencing of snail showed similar enhancement in E-cadherin mRNA levels (Supplementary Fig. 6B). Collectively, these results confirm that CRM1 certainly has a role in promoting snail mediated EMT and that the reversal of EMT by SINE compounds is due in part to suppression of snail especially in the nuclear compartment.

### F-Box protein nuclear retention by SINE compounds induces degradation of snail

**Figure 4:**
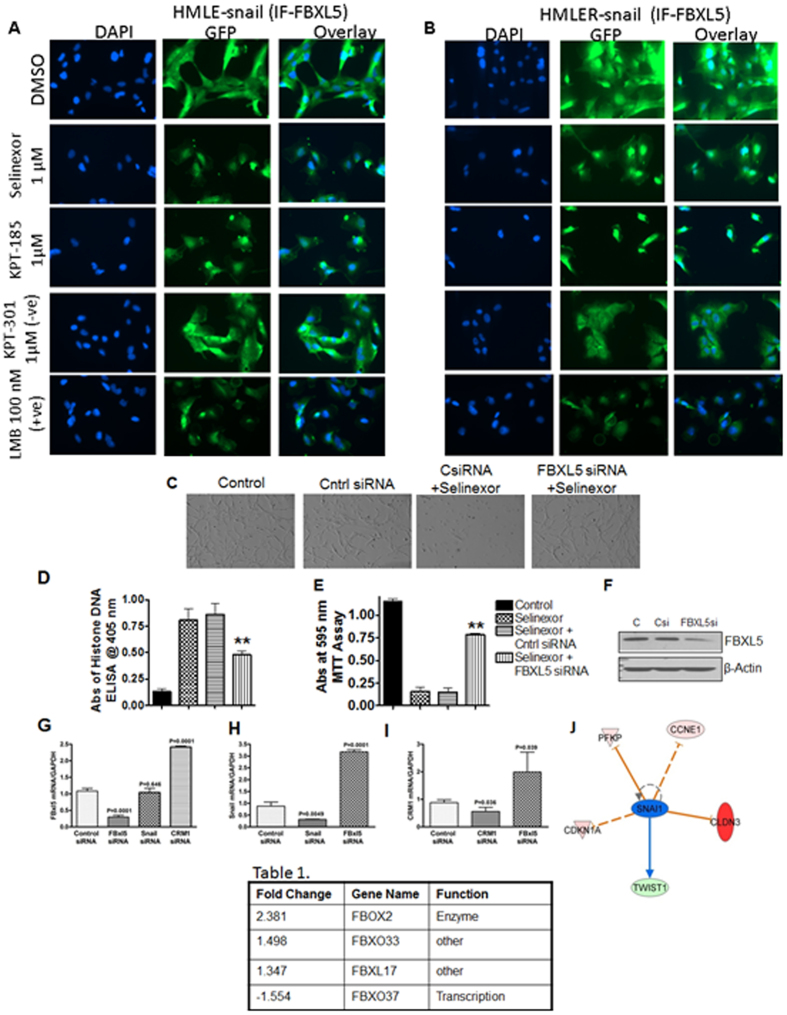
Role of FBXL5 in snail turnover and reversal of EMT. (**A**,**B**) Cells growing in chambered slides (3000 cells per well) overnight and exposed to SINE compounds at indicated concentrations for an additional 24 hrs. Immunofluorescence assay was performed as described in methods section. Immunofluorescence images (40×) of HMLE-snail and HMLER-snail cells showing nuclear retention of FBXL5 (Abcam, USA). (**C**) Photomicrographs of HMLE-snail cells under different treatment conditions. Note. Lack of EMT reversal in the presence of FBXL5 siRNA. (**D**) Histone DNA ELISA apoptosis and (**E**) MTT assay at 72 hrs post Selinexor treatment (150 nM) in the absence or presence of siRNAs for FBXL5 (see methods section for siRNA procedures). **P < 0.01 (siRNA vs untreated groups). (**F**) Western blot showing siRNA silencing results in suppression of total FBXL5. (**G–I**) RT-PCR analysis post FBXL5 siRNA, snail siRNA or CRM1 siRNA silencing respectively. HMLE-snail cells were exposed to FBXL5 siRNA according to procedure described in Methods section. RNA was isolated and RT-PCR was performed. The relative expression of all mRNAs tested by qRT-PCR in control siRNA treated cells were normalized to 1.0 (unit value) and were compared with their respective siRNA treated cells. (**J**) Computational analysis of SINE induced reversal of Mesenchymal phenotype. HMLE-snail cells were seeded in quadruplets in 100 mm petri dishes overnight. Cells were exposed to KPT-185 (150 nM) for additional 24 hrs followed by gene expression microarray analysis (Agilent platform). To construct a p-value for each gene, the test statistics from the 4 replicate experiments were used in a 3 degree of freedom t-test analysis. This t-test is testing whether the average test statistics is not equal to 0 (0 would be no difference treated v. untreated). Each gene thus has a mean log ratio value and a p-value for the detected ratio which determines the significance of the mean log ratio (treated/untreated expression levels combining all 4 replicates). Our objective was to discover what was changed repeatedly in all 4 biological replicates. Statistically significant (p = 0.001) and differentially expressed genes were subjected to Ingenuity Pathway Analysis (IPA) which showed down regulation of Snail network. [Table 1] Some of the F-BOX genes that were differentially expressed (p < 0.001) are tabulated. See Supplementary Table 1 for detailed information on the list of genes differentially expressed.

Recently, it has been shown that F-BOX family member FBXL5 can induce the nuclear degradation of snail^15^. Therefore, in order to validate the role played by FBXL5 in the SINE-induced nuclear degradation of snail, immunofluorescence assays were performed using FBXL5 and snail antibody. Figure 4A,B, shows that 24 hr treatment of HMLE-snail or HMLER-snail cells with 1 μM of either selinexor or KPT-185 or 100 nM LMB, but not –ve control KPT-301, induced the nuclear localization of FBXL5 (marked reduction in the cytosolic compartment). Similar nuclear localization was observed in MCF-7 cancer cells upon SINE (KPT-185) or LMB treatment (Supplementary Fig. 7A). Most significantly, in the presence of FBXL5 siRNA, reversal of EMT morphology by SINE compounds was markedly reduced (Fig. 4C). Furthermore, we observed statistically significant (p < 0.01) reduction in apoptosis (Histone DNA ELISA assay Fig. 4D) and reduction in growth inhibition [p < 0.01 in MTT assay Fig. 4E)] by SINE treatment in FBXL5 silenced cells (Fig. 4F showing western blot for FBXL5 silenced cells), thereby confirming the role of FBXL5. In the presence of FBXL5 siRNA we did not see significant reduction in snail expression upon SINE compound treatment (Figure Supplementary Fig. 7B). Supporting our findings, siRNA silencing of FBXL5 resulted in statistically significant induction CRM1 mRNA levels (Fig. 4G). Snail mRNA levels were slightly enhanced although not in a statistically significant manner. Conversely, siRNA silencing of snail caused statistically significant induction of FBXL5 expression (Fig. 4H). We also explored whether silencing CRM1 could result in the re-expression of FBXL5. In agreement with our hypothesis, CRM1 siRNA treatment of HMLE-snail cells resulted in statistically significant enhancement of FBXL5 mRNA levels (Fig. 4I). Collectively, our results clearly indicate to the role of FBXL5 in the EMT reversal through CRM1 inhibition. In order to further validate our findings, quadruplet RNA samples from KPT-185 exposed HMLE-Snail cells were subjected to Agilent HT12 microarrays followed by pathway analysis. Ingenuity pathway analyses on statistically significant (p < 0.001) and differentially expressed genes (HT12 microarrays) predicted down-regulation of the Snail network (Fig. 4J) [details related to the microarray expression analysis can be obtained at GSE69326 http://www.ncbi.nlm.nih.gov/geo/query/acc.cgi?token=ujwracicfpynluj&acc=GSE69326]. Most significantly, among the major pathways involved, we observed statistically significant changes in F-BOX family members (see Figure 4 inset Table 1 and Supplementary Table 1 for entire set of pathways altered by SINE).

### SINE compounds specificity analysis

**Figure 5:**
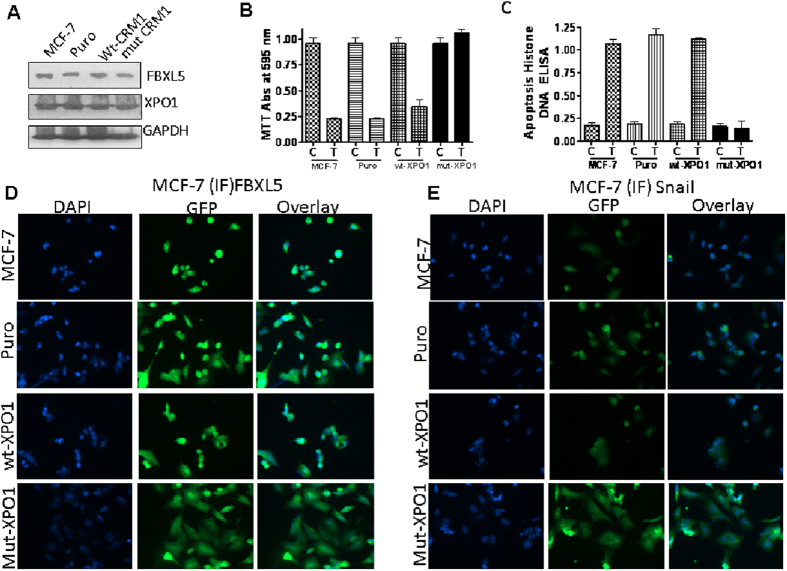
SINE compounds specificity analysis. MCF-7 cancer cells with and without empty vector puro, wild type or mutant-XPO1 (lacking SINE target site Cys528 amino acid. See methods section for transfection procedures) were seeded at 3 × 10^3^ cells per well in 96 well-plates for MTT and 50,000 cells per well for Histone DNA ELISA. After 24 hrs, cells were exposed to 100 nM of selinexor (72 hrs). Cell death was evaluated by MTT and apoptosis by Histone DNA ELISA. (**A**) Protein isolated from different XPO1 construct harboring MCF-7 cells (grown at a density 1 × 10^6^ in petri dish 100 mm size) was subjected to western blotting as described above. Blots were probed for FBXL5, XPO1/CRM1 and loading control β-actin. **Note:** The different cells show normal expression of FBXL5 and XPO1. (**B**) MTT assay. (**C**) Apoptosis. **Note:** Selinexor is ineffective in mut-XPO1 harboring cells (black bars). Graphs representative of two independent experiments (**D**) 3000 cells were seeded in 4 well chambered slides followed by exposure to selinexor at a concentration of 1 μM for 24 hrs as described in methods section. Immunofluorescence images (40×) showing lack of snail degradation or FBXL5 nuclear localization in mut-XPO1 cells. Conversely, snail is found to be degraded in cells harboring wild type XPO1 or empty puro vector (**E**). Images are representative of two independent experiments.

SINE compounds form a covalent bond with Cys528 in the XPO1-cargo recognizing pocket, inactivating XPO1 nuclear export function in a slowly irreversible fashion^21^. In order to validate an XPO1 inhibition-mediated mechanism of action, MCF-7 breast cancer cells were transfected with wild-type or mut-cys528 XPO1 constructs followed by SINE treatment. These cells have normal nuclear export function, express FBXL5 and XPO1 as confirmed by western blotting (Fig. 5A) but the mut-cys528 cells lack the SINE compounds binding site and are resistant to SINE effects^22^. Consistent with this, unlike the wt-XPO1 vector carrying cells, the mut-cys528 cells did not undergo growth inhibition (MTT at 72 hrs Fig. 5B) or apoptosis (Histone DNA ELISA at 72 hrs Fig. 5C). Further, we did not observe either FBXL5 nuclear retention (Fig. 5D) or snail nuclear degradation (Fig. 5E) post treatment in the mut-cys528 carrying cells. These studies confirm the specificity of SINE compounds and support our hypothesis that SINE compounds require XPO1 inhibition through cys528 binding in order to mediate their effects on FBXL5 and snail.

### Selinexor completely suppresses HMCER derived sub-cutaneous xenografts

**Figure 6:**
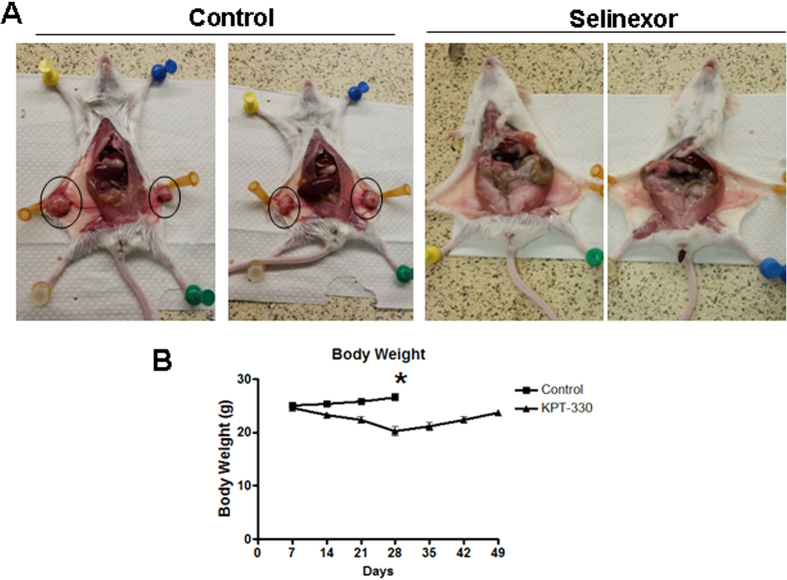
Anti-tumor activity of selinexor in HMLER-snail xengrafts. (**A**) Selinexor treatment results in complete cure of HMLE-Snail xenograft. Mice (n = 6 per group) harboring bilateral tumors were exposed to either vehicle or selinexor at 15 mg/kg orally every other day for three weeks. Left panel showing control mice with tumors and right panel showing selinexor treated mice that are completely devoid of tumors (tumor free at 120 days). (**B**) Mice body weight analysis. *indicates the time point when treatment was stopped and control mice were sacrificed (control tumors reached beyond 2000 mg).

We have previously demonstrated the anti-tumor activity of oral selinexor across a spectrum of sub-cutaneous, orthotopic and systemic tumor models^18^,^23^,^24^. In order to build on these data, we sought to evaluate the anti-tumor potential of selinexor against subcutaneous HMLER-snail xenografts. As shown in Fig. 6A, selinexor administered at 15 mg/kg three times a week for three weeks by oral gavage led to complete elimination of HMLER-snail xenografts. Of greater significance is the observation that we did not observe any tumor regrowth after the end of the three-week treatment period, up to 4 months after treatment when the experiment was terminated. Selinexor was fairly well tolerated at tested dose with acceptable body weight loss that was recovered after the treatment was stopped (Fig. 6B). Collectively, our *in vitro* and *in vivo* results clearly show the EMT reversal capabilities of selinexor, leading to prolonged cures in these subcutaneous xenografts.

## Discussion

In this report we are the first to demonstrate that targeted inhibition of XPO1 by Selective Inhibitor of Nuclear Export (SINE) compound, selinexor can reverse EMT. Selinexor treatment led to complete elimination of tumors in mice. Using molecular and computational analyses, we show that the *in vitro* XPO1 inhibition-induced EMT reversal is directly linked to nuclear localization of F-Box proteins, particularly FBXL5, a protein that is recognized to regulate snail function through nuclear degradation.

The initiation of metastasis, a major cause of treatment failure and morbidity, is believed to be dependent on the aberrant re-activation of the embryonic developmental program called EMT. EMT is a multifaceted biological process that requires a coordinated interaction between numerous signaling pathways. Various EMT regulating TFs are uniquely distributed in different compartments of the cell and their precise location manifests the development of mesenchymal characteristics^25^. Appropriate sub-cellular localization is critical to the function of most of the proteins. TFs regulate the gene expression of various EMT promoters by aligning on DNA in a sequence specific manner for which their nuclear localization is essential. TFs, including notch, snail, wnt/β-catenin, Twist and TGF-β possess conserved nuclear export (NES) and nuclear localization (NLS) signal sequences that allows their recognition by cellular transporters karyopherins that mediate their nuclear-cytosolic shuttling^5^. It is logical to speculate that disturbed nucleo-cytoplasmic transport, as commonly observed in cancer, may result in over-expression of certain EMT promoting TFs.

Snail is one of the most well studied transcription factor and is universally accepted as a driver of EMT. Snail protein is highly labile and is regulated through a precise post-translational ubiquitination control mechanism that is compartmentalization dependent. We had earlier proposed that aberration in nuclear transport mechanisms, particularly in XPO1, may cause a shift in such fine-tuned balance causing the displacement of nuclear proteins, resulting in abnormal signaling mechanisms that may include induction of EMT^5^,^26^. It is logical to assume that targeting the nuclear transport can interfere with this balance leading to modulation of either snail directly or indirectly through proteins that are known to regulate snail function.

F-box proteins, are the substrate-recognizing sub-units of the SCF E3 ubiquitin ligase complexes. These proteins play pivotal roles in multiple cellular processes through ubiquitination and subsequent proteasome-mediated degradation of target proteins^27^,^28^. Dysregulation of F-box protein-mediated proteolysis can lead to malignant transformation in human and other animal cells^29^. Snail is recognized to undergo ubiquitination both in the nucleus as well as the cytosol through a number of F-Box family members. FBXL14 ubiquitinates snail in the cytosol^30^, on the other hand, utilizing a short hairpin RNA screening, it was shown that the FBXL5 protein to be a ubiquitin ligase that can regulate snail protein^15^. This study showed that the cytosolic snail can be forced into the nucleus through CRM1 inhibitor LMB and confirmed the role of CRM1 in snail regulation through transport mechanisms. Although LMB targets XPO1 fairly specifically, the drug could not successfully translate to clinical application due in part of its associated off target toxicity^31^. Small molecule, drug-like SINE compounds (chemically distinct from LMB) that are highly specific for XPO1 with superior pharmacokinetic profiles and therapeutic windows have recently been developed^32^. Selinexor, the clinical stage SINE compound is being evaluated in multiple Phase I and II clinical trials and show single agent activity against a variety of cancers^33^. These multiple lines of evidence clearly demonstrate the superiority of SINE^34^ compounds compared with the natural product predecessor LMB and therefore was evaluated in this study.

We selected the HMECs since EMT induction in these cells is driven by the mesenchymal driver snail making them effective models to evaluate the impact of localization changes that target snail biology. Indeed, SINE treatment resulted in the rapid reversal of EMT (beginning within 3 hours) that was concurrent with nuclear retention of FBXL5 and consequent snail suppression, followed by delayed growth inhibition, and apoptosis. Of importance is the observation that unlike specific snail inhibitors (GN-25) that are required at high micro molar concentrations to achieve IC_50_s in these HMLE cells^35^, the SINE compounds tested here have much higher potency and could induce MET and growth inhibition at much lower concentration range (IC_50s_ < 100 nM) indicating a more efficacious strategy to target EMT by inhibiting nuclear export. Of note, these concentrations are easily achieved at well tolerated doses of selinexor in clinical trials, where Cmax levels in the blood are typically 1.5–2.5 μM.

It is recognized that XPO1 is the major exporter of many nuclear cargoes and not all have tumor suppressor function^36^. The database of the Nuclear Export Signal proteins (NES database) demonstrates that there are more than 200 different export targets of XPO1 including snail (http://prodata.swmed.edu/LRNes)^19^,^37^. One can argue that blocking the nuclear export can retain important oncogenes in the nucleus and this can have adverse effects on normal cells leading to undue toxicity. This could be the reason for premature halting of the single Phase I clinical study involving the XPO1 inhibitor LMB^31^. Given that LMB is a natural product, it is bound to have secondary effects that could be attributed to its toxicity^38^. We do note cell cycle arrest in normal cells by SINE compounds, however, this is transient and the cells proceed to next phase of cell cycle after short halt. On the other hand in cancer cells the cell cycle arrest is followed by apoptosis. Studies from our laboratory and those of others have demonstrated many fold differences in the IC_50_s of cancer and normal cells (pancreas cancer^23^, prostate cancer^39^ and leukemias^40^,^41^,^42^). Unlike LMB, selinexor has been fairly well tolerated and administered in patients for extended periods of time (currently under 43 different Phase I and Phase II clinical studies https://clinicaltrials.gov/ct2/results?term=selinexor&Search=Search). These clinical studies highlight the translational potential of our findings for the treatment of tumors with EMT traits.

We understand that despite this convincing evidence presented in this report, one needs to be cautious in evaluating and interpreting nuclear retention of only a limited set of protein markers by SINE compounds in relation to EMT. This is the reason we undertook a computational approach to better obtain a holistic picture of the different pathways involved in the EMT reversal events. Our pathway analysis show that major EMT promoting pathways are down-regulated by SINE treatment, that we believe could be driving EMT reversal. Highlighting the role of major EMT regulatory pathways, we observed statistically significant changes in multiple F-BOX family members. Among them the FBXO33 has been shown to inhibit multiple TFs including the EMT promoter YB-1^43^ that has consistently been reported to be over-expressed in different tumors^44^. On the other hand FBXL17 has been proposed as a prognostic marker in breast cancer (HMLE-snail are breast cancer derived models)^45^. While RNA interference and site directed mutagenesis studies point to the role of FBXL5 in the reaction, we understand that more work is needed to further substantiate our claims. In this direction, we are currently evaluating SINE activity in HMECs using CRISPR/Cas9 genome editing technology to knockout the F-BOX family proteins that is part of a bigger study that cannot be incorporated in this article. Nevertheless, as revealed by complete cure of the HMLER-snail xenograft models by orally administered selinexor, we strongly believe that targeted inhibition of XPO1-mediated nuclear export could become a novel strategy to target EMT at the nuclear pore.

## Materials and Methods

### Cell lines, culture techniques, and research reagents

HMECs and their subtypes were obtained from Dr. Weinberg, Whitehead Institute, Massachusetts^46^. HMECs were cultured as described in previous publications^47^. MCF-7 cells obtained from ATCC. The cells have been authenticated at the AGTC WSU, (March 13^th^ 2013) using STR profiling by the PowerPlex® 16 System (Promega, Madison, WI). Antibodies for vimentin, snail and E-cadherin, were obtained from Cell Signaling Technologies (Danvers, MA, USA). FBXL5 antibody was obtained from Abcam. The secondary antibodies used in this study were purchased from Sigma (St. Louis, USA).

### SINE Compounds

The SINE selinexor (KPT-330; *in vitro* and orally *in vivo*) KPT-185 (+ve control; *in vitro*) and KPT-301 (inactive *trans-*analog of KPT-185, –ve control *in vitro*] were provided by Karyopharm Therapeutics (Newton MA, USA). Leptomycin B (LMB) was obtained from Sigma (Sigma Aldrich, USA).

### Cell growth inhibition assay (MTT)

5 × 10^3^ HMECs per well were grown in 96-well micro-titer culture plates (Corning, USA). The next day, fresh medium containing SINE compounds was added at indicated concentrations (0–500 nM) diluted from a 1 mM stock solution. After 72 hrs of drug treatment, MTT assay was done through the addition of 3-(4,5-dimethylthiazol-2-yl)-2,5-diphenyltetrazolium bromide ) solution (made by adding 5 mg/mL in PBS) at 20 μL per well for 2 hrs. After 2 hrs, the supernatant was removed and the formazan formed was dissolved (in 100 μL of 2-propanol). The plates were agitated for 30 min, and the absorbance was evaluated using Tecan plate reader (TECAN, Durham, NC) at 595 nm. The IC_50s_ were calculated from the resulting absorbance values using GraphPad Prism 4 software.

### Sphere disintegration assay

HMECs (HMLE-snail and HMLER-snail) in single cell suspension were plated on six well plates [ultra–low adherent wells (Corning, USA)] (density of 1,000 cells/well seeded in sphere formation medium that is composed of 1:1 DMEM/F12 medium and with the supplements B-27-N-2; all obtained from Invitrogen, (Carlsbad USA). The cells were exposed to the different SINE compounds once a week for two weeks and the spheres were counted under an inverted microscope and photographed according to previously described methods^48^.

### Apoptosis Analyses

For apoptosis we utilized the Annexin (Biovision, MA) and Histone DNA/ELISA assay kits (Roche) according to the description provided by the manufacturer. 50,000 cells HMLE-snail and HMLER-snail cells/well grown in six well plates were exposed to SINE compounds at indicated nM concentrations for an additional 72 hrs. After the treatment period was over, the cells were trypsinized, collected and stained with Annexin V and Propidum Iodide. The stained cell population were analyzed using a BD flow cytometer (BD SLR II) at the Karmanos Cancer Institute flow cytometry core. We utilized histone DNA/ELISA as the second assay for apoptosis according to the manufacturers recommended protocol Roche Histone DNA ELISA apoptosis analysis kit (Roche Diagnostics, Indianapolis USA).

### RNA Isolation

HMLE-snail or HMLER-snail cells were grown at a density of 100,000 cells/well in regular six well plates overnight. The cells were exposed to SINE compounds selinexor (1 μM), KPT-185 (1 μM) or LMB (100 nM) for 24 hrs. In a separate experiment, HMLE-snail cells were transfected twice with different siRNAs [control, snail, crm1 or FBXL5 Santa Cruz Biotechnology] for 72 hrs using DharmaFect transfection reagent (Thermo Scientific, Pittsburgh, PA) as described in the manufacturer’s protocol and procedures. Total RNA was isolated using Trizol (Life Technologies) in accordance with the manufacturer’s described protocol. Briefly 1 ml of Trizol was mixed with 200 μl of chloroform and centrifuged at 12,000 × g for 15 minutes at room temp. The upper aqueous phase was mixed with equal amount of isopropanol and centrifuged at a speed of 12,000 × g for 10 minutes. The resulting pellet was washed with 80% EtOH; air dried and was eluted with nuclease free water.

### Quantitative-RT-PCR

We utilized a high capacity cDNA reverse transcription Kit (Applied BioSystems, Foster City, CA, USA) to measure the mRNA level. Approximately 1 μg of RNAs from the different samples was reverse transcribed using 5.8 μl of master mix. The mixture was incubated for 10 min at 25 °C, then 37 °C for another 120 min, and finally 85 °C for 5 min. Quantitative-PCR was performed in triplicate on all samples using SYBR Green PCR Master mix (Life Technologies), cDNA sample. The following primers were used FBXL5: F: 5′ AAGTGGACGTCTTCACCGC, R: 5′ AAGACTGCAGAAGAGCACGG; Snail1 F: 5′ GTTTACCTTCCAGCAGCCCT, R: 5′ TCCCAGATGAGCATTGGCAG; E-Cadherin: F: TGGAGGAATTCTTGCTTTGC: R: CGTACATGTCAGCCAGCTTC. CD24: F: 5′ GCTCCTACCCACGCAGATTT. R: 5′ GCCTTGGTGGTGGCATTAGT using the StepOnePlus RT-PCR (Applied BioSystems). Relative expression of mRNAs was analyzed by utilizing the Ct method and was normalized to GAPDH.

### Western blot analysis

1 × 10^6^ cells were grown in 100 mm petri-dishes over night to ~50% confluence. The cells were exposed to SINE compounds (0–300 nM) or LMB (100 nM) for 72 hrs and protein was isolated. Western blotting was performed as described in detail in our previous publication^48^. For nuclear and cytosolic fraction, the cell pellet was processed using nuclear isolation kit Cayman Chemicals (Ann Arbor, USA).

### siRNA and transfections

All siRNAs were obtained from Santa Cruz Biotechnology (Santa Cruz, USA). Cells were transfected with control siRNA, CRM1 siRNA, snail siRNA or FBXL5 siRNA as described in the manufacturer’s provided protocols^49^. After the siRNA treatments, the cells were photographed under inverted microscope for morphology changes or further exposed to selinexor, KPT-185 or KPT-301 (at IC_50s_) in 96 well plates (5000 cell/well) for MTT and in 6 well plates (50,000 cells/well) for Annexin V FITC assay. The efficiency of the siRNA knock-down was analyzed by RT-PCR and western blotting. CRM1 wild-type and mutant expression constructs and pcDNA3.1-puro empty control plasmid DNA were transfected into cancer cells using Lipofectamine^™^2000 (Life Technologies, Grand Island, NY) by following the recommended procedures. Briefly, one day prior to the transfection, the cells were seeded on 6 well plates in growth medium minus the antibiotics till the cells reached ~90% confluency at the time of transfection. For each sample, 4 μg of DNA was diluted with 250 μl of serum-free medium, and 10 μl of Lipofectamine^™^2000 was also diluted with 250 μl of serum-free medium, mix gently followed by 5 min incubation at room temperature. After incubation period the DNA was diluted with Lipofectamine^™^2000 (total volume = 500 μl) and the mixture was gently mixed and incubated for 20 minutes at room temperature. The reaction mixture (500 μl total) was added to the wells containing cells and medium without antibiotics and the plates were kept in 37 °C in a CO_2_ incubator. Medium was changed after 6 hours. Cells were passaged at a 1:10 dilution ratio into fresh medium lacking antibiotics 24 hours after transfection. Stable cells were selected by the addition of Puromycin at a concentration of 25 μg/mL on the following day.

### Immunofluorescence assay

Cells were seeded on 4 well glass chambered slides (Corning, USA) and exposed to SINE compounds at indicated doses for 24 hrs. After the treatment period was over, the cells were fixed using 4% paraformaldehyde solution for 10 min followed by permeabilization using 0.5% Triton (Sigma-Aldrich, USA). The cells were washed with PBS followed by blocking in 2% BSA solution in TBST for 30 minutes. The blocked slides probed with different primary antibodies overnight at 1:100 dilution and appropriate Alexa Fluor conjugated secondary antibody (1:100 dilution) for 2 hrs. The slides were air dried and mounted using the mounting medium (Gold Antifade mounting medium Invitrogen) and covered with a coverslip and sealed with nail polish. The slides were analyzed under a fluorescent microscope at 40× magnification (Evos FL microscope system).

### Microarrays and pathway analysis

HMLE-snail cells were seeded in quadruplet until they reached ~70–75% confluence. The cells were exposed to KPT-185 (150 nM) for 24 hrs. RNA isolation and quality determination was performed using above described methods. Only the samples having a RIN scores ≥7 were further processed. Whole-genome expression analysis, platform used, pathway analysis and statistics have been described previously^35^.

### Animal xenograft studies

All *in vivo* studies were conducted under Wayne State University Animal Investigation Committee-approved protocol in accordance with the approved guidelines. Four weeks old ICR-SCID mice (female) (Taconic Laboratory) were acclimatized to animal housing facility and xenografts were developed as described in our previous publication^23^. HMLER-snail cells were injected subcutaneously in immunocompromised mice (ICR-SCIDs). To test the efficacy of KPT-330, bilateral fragments of the HMLER-snail xenograft were implanted at the subcutaneous site into 12 mice. One week later, HMLER-snail developed into palpable tumors (~50 mg each) and these tumor-bearing animals were randomized to different cohorts and treated with either diluents (control group), 15 mg/kg of selinexor (given at maximum tolerated dose; MTD) orally every other day, for 3 weeks. All mice were monitored for body weight loss, side effects of the drug treatment and measured for the changes in tumor size.

## Additional Information

**How to cite this article**: Azmi, A. S. *et al.* Targeting the Nuclear Export Protein XPO1/CRM1 Reverses Epithelial to Mesenchymal Transition. *Sci. Rep.* **5**, 16077; doi: 10.1038/srep16077 (2015).

## Supplementary Material

Supplementary Information

Supplementary Video 1

Supplementary Video 2

Supplementary Video 3

Supplementary Video 4
