# Supplementary material for: Targeting the Nuclear Export Protein XPO1/CRM1 Reverses Epithelial to Mesenchymal Transition
Source: Sci Rep. 2015 Nov 5;5:16077. doi: 10.1038/srep16077 (PMC4633607; doi:10.1038/srep16077)
Supplement: Supplementary Information [file srep16077-s1.pdf]

Targeting the Nuclear Protein Export Reverses Epithelial to Mesenchymal Transition

Asfar S. Azmi[1*], Irfana Muqbil[1], Jack Wu[1], Amro Aboukameel[1], William Senapdis[3], Erkan Baloglu[3], Aliccia bollig-Fisher[1], Gregory Dyson[1], Michael Kauffman[3], Yosef Landesman[3], Sharon Shacham[3], Philip A. Philip[1] and Ramzi M. Mohammad[1,2]

Supplemental information

Supplementary Figure 1

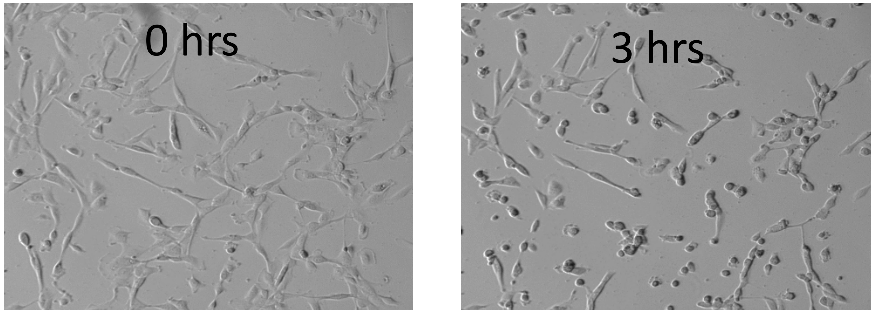

Supplementary Figure 1. Leptomycin B treatment causes reversal of mesenchymal phenotype to epithelial. HMLE-Snail cells were grown in 100 mm petri plates overnight. After 24 hrs the media was aspirated and the cells were washed with PBS followed by incubation with LMB (300 nM) for 3 hrs.

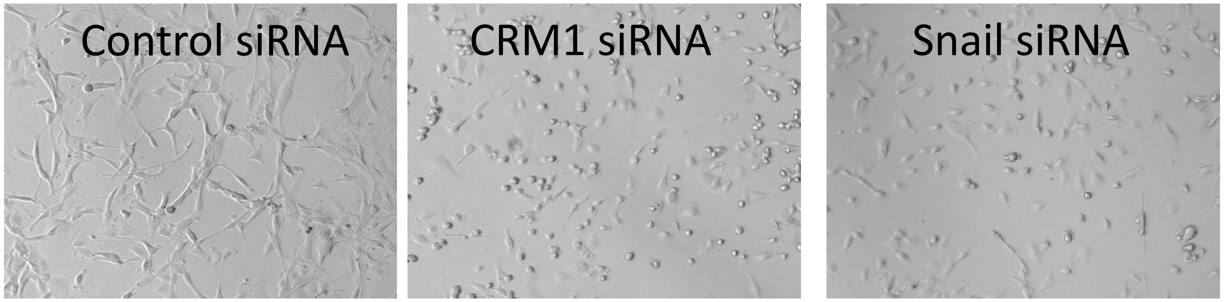

Supplementary Figure 2. siRNA silencing of CRM1 or snail reverses EMT. 50,000 HMLE-Snail cells grown in six well plates in duplicate and were exposed to either control siRNA, CRM1 siRNA or Snail siRNA for 72 hrs. At the end of the siRNA treatment cells were trypsinized and re-seeded and again treated with siRNA for 72 hrs. The cells were photographed at 40X magnification using an EVOS microscope system. Images representative of two independent experiments.

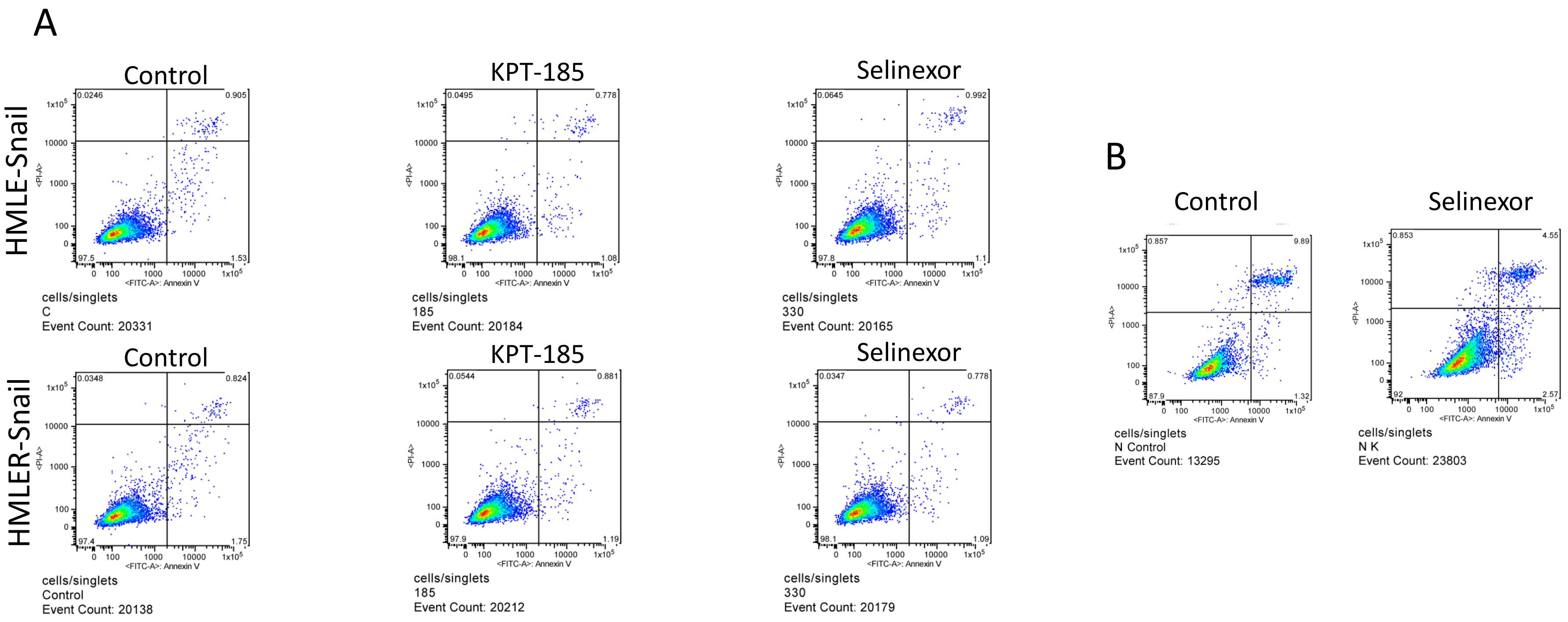

Supp Fig3. SINE do not induce apoptosis at early time points in HMECs. HMLE-Snail and HMLER-Snail seeded at a density of 50,000 cell per well in six well plate were grown overnight. After 24 hrs the cells were exposed to either DMSO or 1 micro M concentrations of SINE (KPT-185 or Selinexor KPT-330) for 24 hrs. At the end of the reaction period, the cells were processed for Annexin V FITC apoptosis analysis using Biovision apoptosis assay kit according to manufacturers protocol. Note: no significant apoptosis is observed at 24 hrs (early) time point. **[B] SINE do not induce apoptosis in MCF-10A NeoT normal cells.** MCF10A-NeoT cells were grown at a density of 50,000 cells per well and exposed to selinexor at 150 nM for 72 hrs. Annexin V FITC assay was performed using Biovision Apoptosis anaylysis kit according to manufacturer's protocol.

Supplementary Figure 3

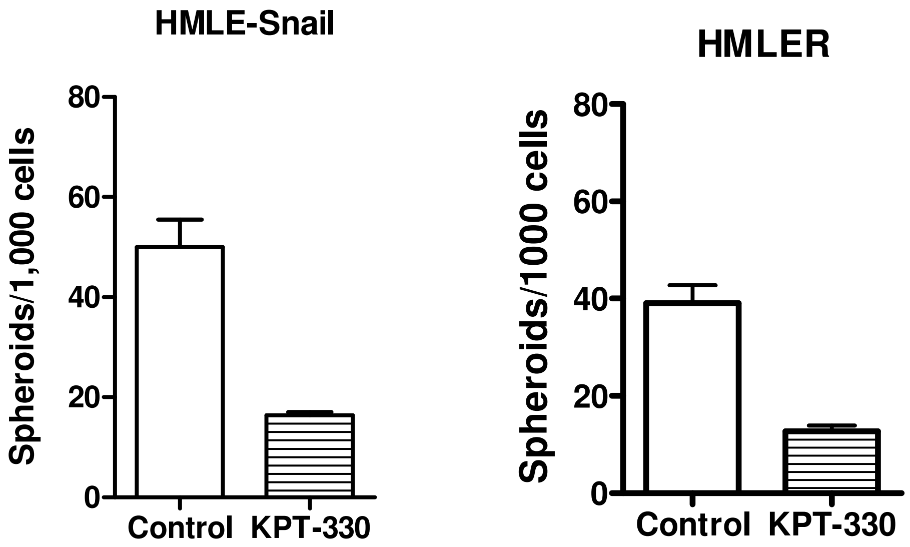

Supplementary Figure 4. Selinexor (KPT-330) suppresses spheroids in HMLE cellular models. HMLE-snail or HMLER-snail spheroids were exposed to selinexor (150 nM) once a week for two weeks. The spheroids were counted under an inverted microscope. See methods section for details on the experimental procedure.

Supplemental Figure 4

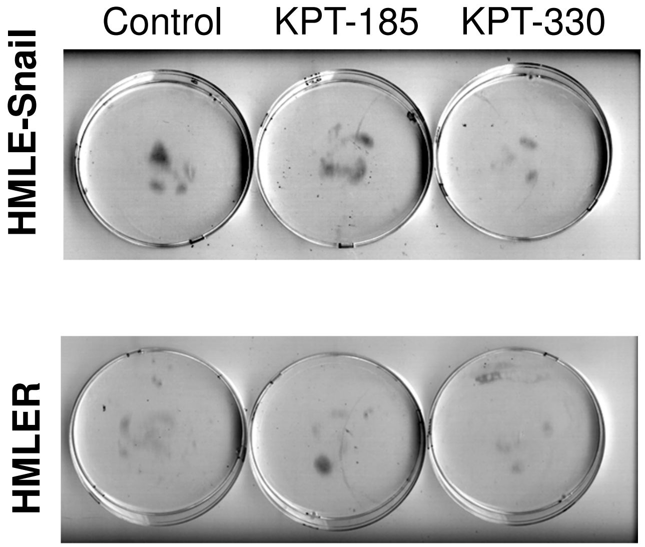

Supplementary Figure 5. SINE suppress colony formation in HMEC derived cells. Clonogenic Assay: 50,000 cells seeded in six well plates and allowed to grow for 24 hrs. Once attached, the cells were exposed to XPO1 inhibitors (0-150 nM) for 72 hrs. At the end of the treatment period, 1,000 cells were taken from each reaction well and re-seeded in 100 mm petri dish and allowed to grow for 2 weeks at 37℃ in a 5% CO_2/5% O_2/90% N_2 incubator. Colonies were stained with 2% crystal violet, counted, and quantitated.
SINE suppress colony formation in HMEC derived cells.

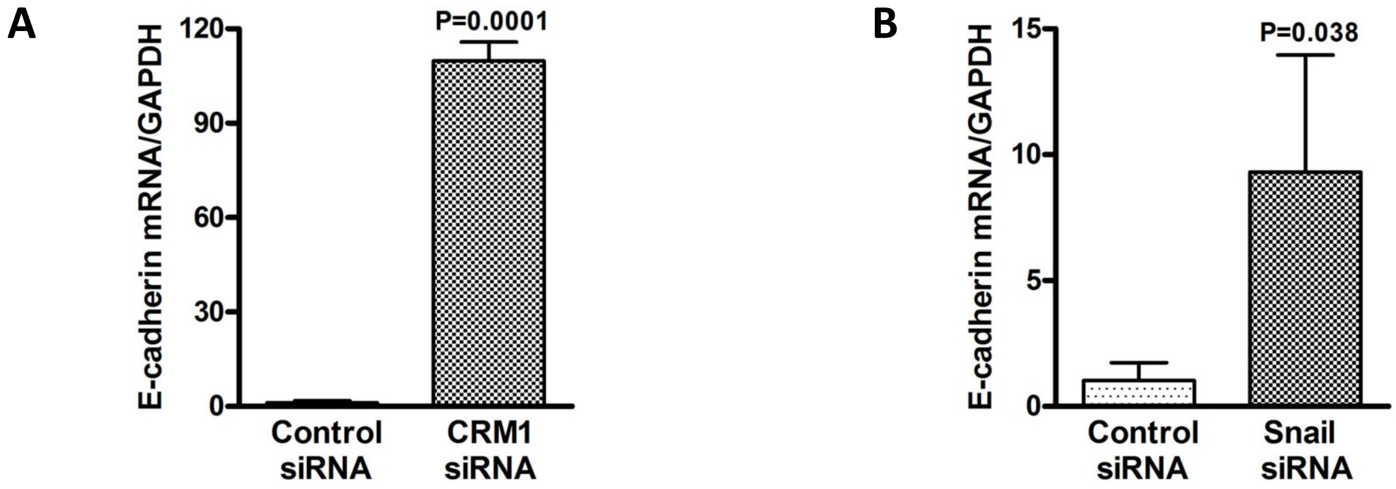

Figure 6. SiRNA silencing of [A] CRM1 or [B] snail enhances the expression of E-cadherin in HMLE-snail cells. HMLE-snail cells were exposed to either CRM1 or snail siRNA and RNA was isolated followed by RT-PCR analysis for E-cadherin expression. Graph representative of two independent experiments.

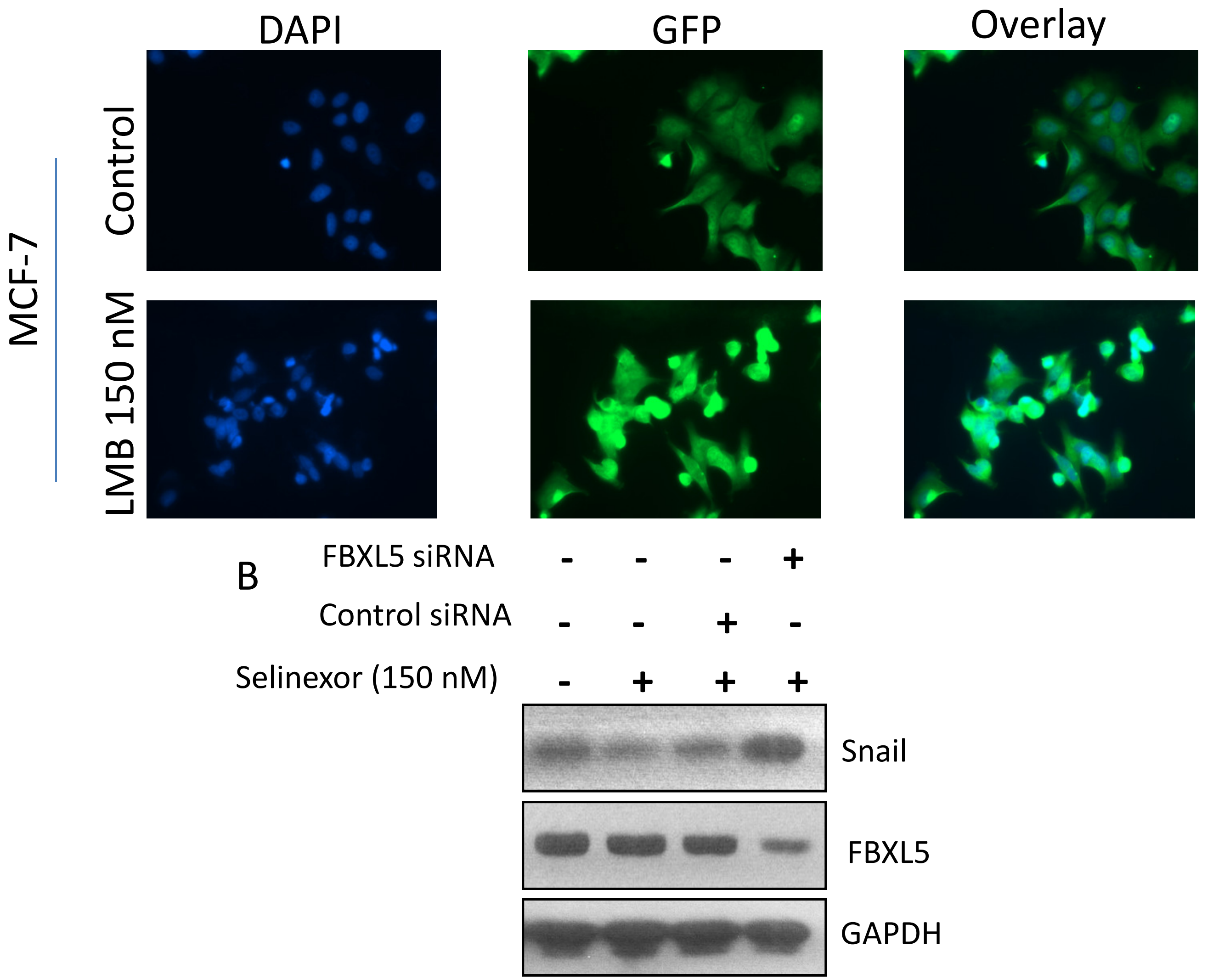

Supplementary Figure 7. [A] LMB induces nuclear localization of FBXL5 in MCF-7 breast cancer cells. Cells were grown in 4 well chambered slides at a density of 3000 cells per well. The next day the cells were exposed to 150 nM LMB for further 24 hrs and immunofluorescence was performed using FBXL5 antibody. **[B]** Western blotting of HMLE-Snail exposed to either untreated, control siRNA or FBXL5 siRNA in the presence or absence of selinexor 150 nM. Note: lack of snail reduction upon FBXL5 siRNA treatment Note. Nuclear retention of FBXL5 in treated group. Figure representative of three independent experiment.

Supplementary Table 1. FBOX family genes are differentially expressed in SINE treated cells and fall under the canonical biologically meaningful pathways

Network	Molecules in Network	Focus Molecules	Top Functions
1	ABAT,ATP2B2,BBS9,CASK,CTH,Cytokeratin,DDIT3,DDX56,ERK,**FBXO33**,HES4,IPO5,KIF5C,KRT7,KRT19,KRT6A,Laminin1,LARGE,MT1B,NELF,PCM1,Pmca,POLR1B,PSAT1,Ribosomal 40s subunit,Rnr,RPL13,RPL37,RPS24,RPS15A,RPS3A,RPS6KA,RPSA,RRS1,TRIB3	28	Gene Expression, Protein Synthesis, Cancer
2	ABCC1,ABCD3,Actin,AIMP2,ANXA3,ATPase,BCCIP,Cbp/p300,CCNK,E2F5,EIF6,EPAS1,FAM60A,GRN,GTF2A1,HAUS6,HIRIP3,HIST1H2BJ/HIST1H2BK,Holo RNA polymerase II,HSPA4L,KIFC2,NHP2L1,NMI,NUDT5,P-TEFb,Rac,RNF213,RUVBL1,SNRNP48,snRNP,Sos,TAF9,TUFT1,tyrosine kinase,ZCRB1	26	DNA Replication, Recombination, and Repair, Energy Production, Nucleic Acid Metabolism
3	ANGPTL4,BMP10,BMP,BNC2,C8orf33,CLGN,EDA,FST,FUCA1,GFPT2,GLA,GLRX,IgG2a,IgG2b,IL13RA2,IL18BP,IL18R1,IL23A,INF2,IRAK2,IRAK,IRF5,ITM2B,Mhc class ii,NFkB (complex),PCSK5,PPP4R4,RIPK4,SAA,SERCA,SLC11A2,Smad,SPPL2A,TIRAP,ZFAND5	26	Inflammatory Response, Organismal Injury and Abnormalities, Cellular Function and Maintenance
4	BDNF,Calmodulin,CASP3,caspase,CLEC4D,CLIC4,CLN8,CNNM4,DNAJC8,DNAJC9,DNAJC15,DNAJC,DYNC1I2,estrogen receptor,FEM1B,FOXM1,HNRNPU,Hsp27,Hsp70,Hsp90,Hsp22/Hsp40/Hsp90,IGF2,KANK2,LYN,NMDA Receptor,NOS2,p70 S6k,Proinsulin,RNF103,S100A4,SEZ6L2,Sod,TIMM13,UNC5B,VEGFA	23	Cell Death and Survival, Nervous System Development and Function, Cellular Movement
5	Alp,ALT,BRD2,CCDC71,CDKN1A,Ck2,COG1,COG5,DDB2,DKK1,DPAGT1,DZIP1,FCRLA,GOT,Histone h3,IgG1,IgG,Igm,Immunoglobulin,Insulin,KAT2B,MTOR,NFE2,PBX3,PFKP,PHEX,RAB17,RNA polymerase II,SAP130,SIGLEC5,SIX1,STAT5a/b,Tnf (family),TYMS,UBQLN1	22	Developmental Disorder, Hereditary Disorder, Metabolic Disease
6	26s Proteasome,Alpha tubulin,ANKRD28,Beta Tubulin,CALCOCO2,CCDC28A,CCT3,CCT5,CLDN3,ELMOD3,F Actin,FAM90A1,HDAC6,HIPK2,HISTONE,Ikb,LZTS1,MGEA5,NEXN,NFKBIB,NFKBIE,PCDH18,Pka,PP2A,PRKAR1B,RASGRP2,RUNX2,Smad1/5/8,SYNE2,TAGLN,Tnf receptor,TPPP,Troponin t,Tubulin,Ubiquitin	22	Cardiovascular Disease, Cell-To-Cell Signaling and Interaction, Cellular Movement
7	APOBEC3B,BCL2A1,Caveolin,CES1,CLU,CLUAP1,endothelin receptor,Ferritin,Fibrinogen,FIP1L1,FOS,FOSB,FTH1,FTL,glutathione transferase,GST,GSTA4,GSTM4,hemoglobin,HMOX1,IFITM1,IL11,Integrin alpha V beta 3,Ldh,LDL,MLF1,NAB1,NAB2,NADPH oxidase,Pde,PDE7B,RGMA,SOX17,Stat3-Stat3,TSH	21	Cellular Development, Cellular Growth and Proliferation, Connective Tissue Development and Function
8	20-alpha-hydroxyprogesterone,AES,APP,ATP2B2,BCAS1,beta-estradiol,CC2D1A,CCDC88B,CGB (includes others),CLDN3,DNAH14,DNALI1,ECH1,GJB2,HRK,HSD17B2,ITGBL1,NCAPG,NUDT9,OSBPL5,PPM1K,PPP1R14A,progesterone,PTGFR,RASGRP2,RER1,RYR3,SERPINA6,SH3GL3,SIX4,SLC30A3,SLC7A2,SPAG9,SULT1E1,TCF15	22	Reproductive System Disease, Cell Death and Survival, Nervous System Development and Function
9	ABCA7,Akt,atrial natriuretic peptide,CAV3,creatine kinase,CREG1,CTC1,cyclooxygenase,Foxo,FXR ligand-FXR-Retinoic acid-RXRα,Growth hormone,HDL,HDL-cholesterol,Kallikrein,LDL-Cholesterol,LPXN,MYL9,Nos,NR4A3,OBFC1,PCSK9,PCYOX1,PDK4,Ppp2c,PRKAR2B,PVRL2,SCARB1,SEC14L2,SGMS1,SH2B3,SLC9A3R1,SOAT2,SYNM,VLDL-cholesterol,VLDLR	21	Lipid Metabolism, Molecular Transport, Small Molecule Biochemistry
10	ADCY,ADORA2B,ADRB,Ampa Receptor,CHRM4,DUB,EDNRA,FAF2,G protein alpha,G protein alphai,GABBR2,Gi-coupled receptor,Girk,GPER,Gs-coupled receptor,GTPase,LAPTM5,LPAR1,MRPL20,MTORC2,NPR3,OAZ1,Pkc(s),Pkg,PLC,Plc beta,PLCB2,RGS4,RHEB,SLC30A3,UBR2,ULK2,USP13,USP47,USP48	20	Carbohydrate Metabolism, Molecular Transport, Small Molecule Biochemistry
11	Ap1,ATXN10,BAMBI,BTG2,calpain,CaMKII,CCNE1,CENPI,Cg,Creb,EDN1,FJX1,FSH,GLS,Gsk3,Histone h4,HMBS,HSD3B7,IL1,JPH4,Lh,LRP,MCM7,NOS1AP,Notch,NRARP,PDGF BB,PDLIM3,PPARG,Pro-inflammatory Cytokine,PTX3,SLC7A7,SOCS2,SRRT,Tgf beta	20	Organismal Functions, Cell Morphology, Renal and Urological System Development and Function
12	AMPK,ASNS,ATP6V1C1,c-Src,Cdc2,CDH18,Cdk,Cyclin A,Cyclin D,Cyclin E,cytochrome C,E2f,FAHD1,FANCG,HMG CoA synthase,HOPX,HOXB9,HPSE,INA,KCNMA1,MPC1,MTORC1,NPC1,NT5C2,OR7C2,PARP,PIK3R3,PPAP2A,PPAP2B,PRKAG2,Rb,SAT1,Secretase gamma,trypsin,Vegf	19	Cancer, Reproductive System Disease, Carbohydrate Metabolism
13	AFAP1L2,ARHGDIB,ATF3,CHRNA7,Collagen Alpha1,Eotaxin,Ern,**FBXO2**,Fcer1,Fgf,FGFR3,Fgfr,FRMD3,Gap,GJB2,Hedgehog,HES6,HHIP,Hspg,IGSF8,IL4R,JAK,MGAT5,MMP1,MT2A,Nicotinic acetylcholine receptor,Patched,PI3K (complex),PLC gamma,PRRX1,STAT1/3/5 dimer,SYK/ZAP,Tenascin,TIMP3,TWIST1	18	Cellular Growth and Proliferation, Cell Signaling, Connective Tissue Development and Function
14	ARHGEF39,C2orf43,COMMD1,DPY19L1,DVL2,ELAVL1,EMC7,FAM136A,GINM1,HKDC1,KCTD14,KDM5B,KIAA0922,LMNA,LRRC37B,LSM1,MIOS,PKD1,RAE1,SCNN1D,SLC22A23,SMARCA4,SUCO,SYVN1,TMEM38B,UBC,VSIG10	16	Cellular Assembly and Organization, Organ Morphology, Reproductive System Development and Function
15	14-3-3,ABLIM1,ADAMTS1,ARC,BCL2L11,BCR (complex),BPGM,Calcineurin protein(s),CD302,CDA,DHRS9,Ecm,EGR1,EGR2,EGR4,Hdac,Iga,IL7R,Importin alpha,L-type Calcium Channel,MAP2K1/2,methenyltetrahydrofolate cyclohydrolase,MME,MTHFD1,MTHFD1L,MTHFD2L,Nfat (family),OSR1,RAB3IP,Raf,Ras,RUNX1T1,Sapk,TCR,TXNRD2	20	Cellular Compromise, Organismal Injury and Abnormalities, Cell-mediated Immune Response
16	Adaptor protein 1,Caspase 3/7,CFL1,CLCN5,Cofilin,FGD4,GCNT1,GKAP1,Gm-csf,GPS2,HNRNPA3,IRS,Jnk,JUN/JUNB/JUND,KCNJ15,LAMP3,LHX1,Mek,Mlc,MSI2,Myosin,NRG (family),PAK6,Pak,PP1 protein complex group,RBM47,Rock,RPS6KA2,Rsk,SESN2,SHANK1,SMPD4,sphingomyelinase,ST6GAL1,TESK2	19	Cellular Assembly and Organization, Hereditary Disorder, Neurological Disease
17	BHLHE40,C/ebp,CIITA,CXCL14,CXCR3,EIF3,EIF4G3,elastase,HEXB,HLA-DR,HLA-DRB5,HSP,IFI44L,IFIT1,Ifn,IFN alpha/beta,IFN Beta,Ifn gamma,IFN type 1,IFNA21,Ige,IL12 (complex),IL22RA1,Interferon alpha,MHC Class II (complex),NfkB1-RelA,P38 MAPK,PCDH7,PI3K (family),PLP1,SEC14L3,SERPINB9,Tlr,TNFSF9,TRIP10	17	Cellular Function and Maintenance, Hematological System Development and Function, Humoral Immune Response
18	ABCA6,AOX1,BATF2,BATF3,BCL2A1,CACNB3,Cbp/p300-Maf-Nfe2l2,CEBPD,CYB561,EIF2AK4,FGF1,GCLM,GSTA4,GSTA5,GSTP1,HCP5,IFNG,IKZF2,IL11,KRTAP2-3 (includes others),neuroprotectin D1,NFE2L2,NFE2L3,PRICKLE1,RAB34,SHISA5,SLC14A[illegible],[illegible],SLC6A1,SLC7A11,SMAD4,TBXAS1,TCTA,TRIM33,UGT2B15	17	Drug Metabolism, Protein Synthesis, Cellular Assembly and Organization
19	BABAM1,BRD4,CLN3,COMMD1,COMMD7,COPS6,DIS3L2,**FBXL17**,FEZ1,G3BP1,HERC4,HERC6,KBTBD7,MLH1,MRPL45,MYADM,NME2,PDX1,PRRC1,RAD18,RTN4,SEL1L3,SELRC1,SFXN4,SLC25A23,SPRTN,TAGLN3,TLN1,TRMT6,TRMT61A,TRNT1,TUBB3,TUBB4A,UBC,ZNF14	16	Cancer, Hereditary Disorder, Renal and Urological Disease
20	AKNA,AKR1C3,ARL1,ARMC8,CENPN,CENPO,CHAC1,CUTA,FAM213B,GCC2,GID8,GIGYF2,GNE,GNPNAT1,IQCB1,KNTC1,MAEA,MKLN1,NAGK,NBAS,NPHP4,RALGAPA1,RALGAPB,RINT1,RMND5A,RPGRIP1L,SCFD2,SEMA5A,TBRG1,UBC,UBXN8,WASF2,YIF1A,ZBTB38,ZWILCH	16	Carbohydrate Metabolism, Small Molecule Biochemistry, Hereditary Disorder
21	ACADSB,AP4B1,AP4M1,AP4S1,CHST12,CLASP1,CYP4F3,CYP4F11,DDX52,GCLM,GFM1,H2AFV,LRBA,LSR,MCTS1,MEF2B,PIGH,PIGQ,PIGY,PPHLN1,PSMF1,PYCR1,RAB10,RPL17,SEPT8,SLC35B2,SRSF7,STOX1,TBC1D7,TGOLN2,UBC,UQCR10,UQCR11,UQCRB,WDR41	16	Hereditary Disorder, Neurological Disease, Metabolic Disease
22	ADCK1,ANXA8L2 (includes others),ARHGEF26,ASPM,C11orf96,CHML,CYB561,DARC,ESM1,FLT3LG,GAS1,GCNT1,GLI1,GPER,HSPB2,hydroxyproline,ICAM1,IL17F,Integrin alpha V beta 3,MAN1C1,NREP,OVOL1,PDLIM7,PKIG,PLOD1,RAB1A,RAB6A,RNF152,S1PR3,SCG5,SLC13A3,TGFB1,TLR5,TMEM100,TNFSF13B	16	Cell-To-Cell Signaling and Interaction, Cellular Movement, Hematological System Development and Function
23	ADAM32,C1orf74,CEP19,CLDN1,DHX8,DNAJC30,EXOSC2,FAM107B,GATA4,GCKR,GOT1,GPR39,GRHPR,HNF1A,HNF4A,HPX,HSD17B2,HSPA4L,IER5,ITIH4,KIAA0101,LAS1L,MLF1IP,NUF2,REPIN1,RPS25,RRP8,SLC26A1,SLC37A4,SLC38A4,SPCS3,TBX18,UGT2B15,ZNF443,ZNF576	16	Carbohydrate Metabolism, Molecular Transport, Small Molecule Biochemistry
24	ATM,CCND1,CCR6,CD3,CDX1,DEF6,DICER1,eIF2B,EPSTI1,FAM129A,FAM65B,ICOS,IL2,IL-2R,IL12RB1,IL18R1,IL23R,IL7R,KRT80,LINC00667,miR-19b-3p (and other miRNAs w/seed GUGCAAA),MSLN,NANOS1,NEAT1,PIK3R3,PRKAR2B,PTPN22,RNF139,SATB1,SIPA1L2,SYAP1,Tcrd,TMEM117,TMSB15A,TNFRSF4	16	Cell-mediated Immune Response, Cellular Development, Cellular Function and Maintenance
	Alpha Actinin,Alpha catenin,ANTXR2,COL17A1,COL1A1,COL1A2,COL3A1,COL8A1,collagen,Collagen type I,Collagen type III,Collagen type IV,Collagen [illegible]		

Legends to supplementary videos 1, 2, 3 and 4

Supplementary video 1. HMLE-snail cells were grown at a density of $1x10^6$ in 100 mm petri dish overnight. After 24 hrs the media was aspirated and the cells were washed with PBS followed by incubation with DMSO for 3 hrs with simultaneous live video recording at 40 X magnification under a EVOS FL microscope system.

Supplementary video 2. HMLE-snail cells were grown at a density of $1x10^6$ in 100 mm petri dish overnight. After 24 hrs the media was aspirated and the cells were washed with PBS followed by incubation with selinexor (KPT-330) for 3 hrs with simultaneous live video recording at 40 X magnification under a EVOS FL microscope system.

Supplementary video 3. HMLE-snail cells were grown at a density of $1x10^6$ in 100 mm petri dish overnight. After 24 hrs the media was aspirated and the cells were washed with PBS followed by incubation with KPT-185 for 3 hrs with simultaneous live video recording at 40 X magnification under a EVOS FL microscope system.

Supplementary video 4. HMLE-snail cells were grown at a density of $1x10^6$ in 100 mm petri dish overnight. After 24 hrs the media was aspirated and the cells were washed with PBS followed by incubation with LMB (500 nM) for 3 hrs with simultaneous live video recording at 40 X magnification under a EVOS FL microscope system.
